# How should painful cystic degeneration of myomas be managed during pregnancy? a case report and review of the literature

**Published:** 2011

**Authors:** Tae-Hee Kim, Hae-Hyeog Lee

**Affiliations:** Department of Obstetrics and Gynecology, College of Medicine, Soonchunhyang University, Bucheon, 420-767, Republic of Korea.

**Keywords:** *Leiomyomas*, *Uterine Fibroids*, *Pregnancy*

## Abstract

**Background:**

Uterine myomas are common pelvic masses during pregnancy. The pain and rapid growth of myomas are among the most common complications during pregnancy. We evaluate management of painful cystic degeneration of myomas during pregnancy.

**Case:**

A 27-year-old primigravida had a pelvic mass. We have managed a case in which the diagnosis of cystic degeneration of uterine myomas could not be easily differentiated from an ovarian torsion or carcinoma. Differentiation between degenerative pain of the myoma and an ovarian malignancy or torsion was necessary. A complete aspiration of the cystic changes of the uterine myoma was performed without performing a myomectomy.

**Conclusion:**

We report a good result of aspiration of a cystic uterine myoma during pregnancy with a review of the literature published for twenty years since 1 January 1988.

## Introduction

Uterine leiomyomas are observed in pregnancy more frequently now than in the past because many women are delaying childbearing. Uterine myomas during pregnancy increase the incidence of spontaneous abortions, ectopic pregnancies, preterm labor, premature rupture of the membranes, placental abruptions, abnormal fetal presentations, and red degeneration. Myomectomy during pregnancy is rarely performed because of a fear of pregnancy loss and bleeding (1). We report a case of an aspiration of a painful uterine myoma during pregnancy.

## Case Report

**Figure 1:**
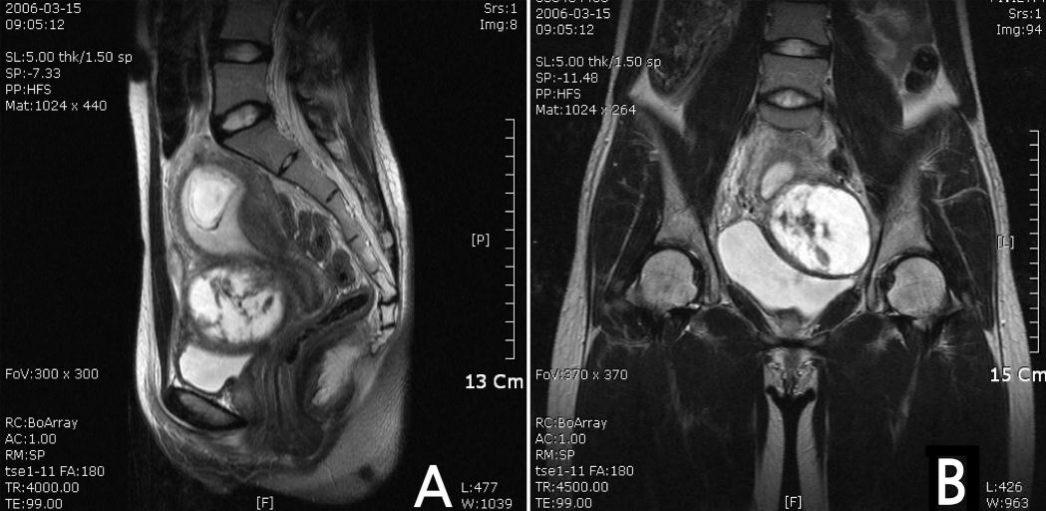
T2-weighted image of MRI findings showed an 8×7×6 cm cystic and solid mass with septa. (A) Sagital view, (B) Coronal view.

We performed a cystic myoma aspiration at 12 weeks gestation with preservation of the pregnancy. A 27-year-old primigravida had an 8 cm pelvic mass on routine ultrasonography at 10 weeks gestation. She had a MRI at a local clinic. The MRI demonstrated an 8 x 7 x 6 cm cystic and solid mass with septa (Figure 1). The presumptive diagnosis was a cystic borderline tumour or cyst adenocarcinoma. She was admitted to the emergency room for pain management. Differentiation between degenerative pain of the myoma and an ovarian malignancy or torsion was necessary. We performed explolaparotomy. Intra-operatively, the uterus was soft and the size was appropriate for 12 weeks gestation. The leiomyoma was situated in the broad ligament at the left anterior aspect of the uterus. The left ovary was located on the myoma of the broad ligament. We performed a complete aspiration of the cystic changes of the uterine myoma, washing cytology, and biopsy of the cyst wall of the uterus without performing a myomectomy. The washing cytology showed no malignant cells; the biopsy specimen was a uterine leiomyoma. After cystic aspiration, the pain was relieved. There were no other complications during pregnancy. She admitted for 10 days for fetal monitoring and postoperative care after explolaparotomy. She had no fever, complication and preterm labor during pregnancy. She had a myomectomy during caesarean section and a healthy female weighing 2, 640 grams at 38 weeks gestation. Three years later, she had another healthy baby and there were no other complications. 

**Table I:** Clinical characteristics of patients in articles including our case

	**Age** **(years)**	**Parity**	**Gestational age (week)**	**Diagnostic method**	**Myoma** **size (cm)**	**Symptoms**	**Delivery mode**	**Delivery time** **(weeks)**	**Newborn** **weight** **(g)**
Michalas 1995 (2)	31	primi.	14	USG	23 cm	respiratory pain	C/S	39	2000 g
Mollica 1996 (3) (18 cases)	33 (28-40)	primi.16 multi. 2	12 (10-19)	NM	5-10 cm; 9 cases 10 cm< ;9 cases	pain rapid growth	C/S 93.7%	N.M	>2500 g; 17 cases <2500 g; 1 case
Majid 1997 (4)	35	primi.	17	USG	24 cm	nausea and vomiting	C/S	17^+5^	NM
Ehligiegba 1999 (5)	28	NM	28	NM	6 cm	umbilical hernia	V/D	39	3400 g
Wittich 2000 (6)	31	primi.	11^+4^	MRI	2074 g	pain	C/S	NM	3275 g
Danzer 2001 (7)	44	primi.	12	USG	10 cm	pain, bleeding	C/S	37	3235 g, 2810 g
Carolis 2001 (8) (18 cases)	21-34	14-primi. 4-multi.	15.1±5.0	NM	14.3 ±7.9 cm	pain-8 cases pyrexia-4 cases	C/S : 14 V/D : 2	37.6±4	3273.7 g ±399.3
Celik 2002 (9) (5 cases)	31.4±3.5	primi.:2 multi.:3	17.8±3.4	NM	14 ±3.8 cm	pain	C/S:5	38.6±1.1	3220 g ±303.3
Lolis 2003 (10) (13cases)	32.9±5.0	NM	16.2±1.3	NM	879.5 g±52.9	pain:10 cases increase size:3 cases	C/S	37.1±2.7	3048.4 g ±528.2
Melgrati 2005 (11)	29	primi.	24	USG	7 cm	pain, fever	V/D	39	NM
Umesurike 2005 (12)	30	primi.	20	USG Cyst	32 cm 7700 g	pain	V/D	38.9	3.560 g
Usifo 2007 (13)	31	primi.	13	USG Cyst	2000 g	pain, nausea, vomiting, diarrhea	C/S	38	3990 g
Bonito 2007 (14) (5cases)	34.4		11.8	NM	7 cm	pain, rapid growing	C/S:2, V/D:3	38.2	3340 g
Okonkwo 2007 (15)	41	multi.	19	USG	1000 g	free fluid in abdomen	C/S	38	2600 g
Bhatla 2009 (16)	30	primi.	19^+3^	USG	3900 g	pain	V/D	38	2740 g
Our case 2009	27	primi.	12^+2^	MRI	8x7x6 cm	pain	C/S	38	2640 g

Primi= primi-parity, multi= multi-parity, NM= not mention, USG= ultrasonogram, MRI= magnetic resonance images, C/S=caesarean section, V/D=vaginal delivery.

## Discussion

The PubMed search engine was used to identify Korean and English language articles published between 1 January 1988 and 1 January 2009 using the search terms myomectomy, cyst aspiration, and pregnancy. We found 18 articles, 2 of which could not be accessed outside the web in Korea. In 71 cases, including the case herein, operative procedures of myomas during pregnancy were performed (2). The clinical characteristics of published patients, including the patient presented herein, are listed in table I (2). Our case had features of an ovarian tumor or torsion similar to 5 other cases.

Uterine leiomyomas occur in 1.6% ~ 2% of pregnancies (16). Although leiomyomas during pregnancy usually remain asymptomatic, they may have complications. The most common complication of uterine myomas during pregnancy is abdominal pain, which is due to red or carneous degeneration.The management of painful leiomyomas during pregnancy is usually medical, but a few myomectomies have been reported. Myomectomies are generally avoided during pregnancy because increased vascularity of the uterus can lead to hemorrhagic complications. Successful myomectomies during pregnancy have been reported. The cases showed that the most common indication for myomectomy during pregnancy was severe abdominal pain to maintain pregnancy without any procedure. In the article review, there were gastrointestinal manifestations such as nausea, vomiting and diarrhea due to obstructive pressure of myomas on bowel. When we have cases which require myomectomy during pregnancy, addition of Doppler evaluation is 

recommended. A sharp drop in residence index (RI) in Doppler means an indication of some degree of necrosis (17). The Doppler is a helpful modality to decide doing myomectomy or not during pregnancy (17). In 71 cases, only 2 pregnancies were terminated after myomectomy (4, 8) and 2 cases had preterm labor and preterm delivery respectively (3, 10). One case had intrauterine growth retardation (4). There was one prospective large study. Among 15, 579 women registered at the prenatal clinic, severe abdominal pain was seen in 16 patients, in 13 cases myomectomy was done. 12 cases has live birth, 13 cases has no blood transfusion and other complications (10).

There has been only one successful case of a gasless laparoscopic myomectomy (11). In this case, it could be difficult to differentiate a complex ovarian mass from cystic degeneration of the myoma. The prevalence of adnexal malignancy in pregnancy is between 2% and 3% (18). The likelihood of malignancy with a complex ovarian mass is relatively low, but one must consider surgery during pregnancy, and routine prenatal ultrasound (US) to detect an ovarian mass. Magnetic resonance imaging (MRI) can be safely used during pregnancy to evaluate adnexal masses. But only one case was evaluated by MRI. The severe abdominal pain during pregnancy could be considered to perform explolaparotomy or not. Degeneration of a myoma may mimic an ovarian malignancy during pregnancy and obstetricians have to be aware of the differential diagnosis.

Our report provides suggestions for obstetricians on the management of symptomatic uterine myomas during pregnancy. A myomectomy during pregnancy in carefully selected patients is a safe procedure. 

From the literature review, there were rare cases of cystic degeneration of uterine myomas during pregnancy. 

The severity of symptoms and suspicion of malignant mass or torsion is the key in deciding upon laparotomy. We recommend to perform cyst aspiration rather than myomectomy in a myoma with cyst degeneration and pain.
